# Bacterial Diversity and Community Structure of Supragingival Plaques in Adults with Dental Health or Caries Revealed by 16S Pyrosequencing

**DOI:** 10.3389/fmicb.2016.01145

**Published:** 2016-07-22

**Authors:** Cuicui Xiao, Shujun Ran, Zhengwei Huang, Jingping Liang

**Affiliations:** Shanghai Key Laboratory of Stomatology, Department of Endodontics and Operative Dentistry, Ninth People's Hospital, School of Medicine, Shanghai Jiao Tong UniversityShanghai, China

**Keywords:** dental caries, bacterial diversity, dental plaque, pyrosequencing, 16S rDNA

## Abstract

Dental caries has a polymicrobial etiology within the complex oral microbial ecosystem. However, the overall diversity and structure of supragingival plaque microbiota in adult dental health and caries are not well understood. Here, 160 supragingival plaque samples from patients with dental health and different severities of dental caries were collected for bacterial genomic DNA extraction, pyrosequencing by amplification of the 16S rDNA V1–V3 hypervariable regions, and bioinformatic analysis. High-quality sequences (2,261,700) clustered into 10,365 operational taxonomic units (OTUs; 97% identity), representing 453 independent species belonging to 122 genera, 66 families, 34 orders, 21 classes, and 12 phyla. All groups shared 7522 OTUs, indicating the presence of a core plaque microbiome. α diversity analysis showed that the microbial diversity in healthy plaques exceeded that of dental caries, with the diversity decreasing gradually with the severity of caries. The dominant phyla of plaque microbiota included *Bacteroidetes, Actinobacteria, Proteobacteria, Firmicutes, Fusobacteria*, and *TM7*. The dominant genera included *Capnocytophaga, Prevotella, Actinomyces, Corynebacterium, Neisseria, Streptococcus, Rothia*, and *Leptotrichia*. β diversity analysis showed that the plaque microbial community structure was similar in all groups. Using LEfSe analysis, 25 differentially abundant taxa were identified as potential biomarkers. Key genera (27) that potentially contributed to the differential distributions of plaque microbiota between groups were identified by PLS-DA analysis. Finally, co-occurrence network analysis and function predictions were performed. Treatment strategies directed toward modulating microbial interactions and their functional output should be further developed.

## Introduction

The microorganisms that live in and on the human body are estimated to outnumber human cells by at least an order of magnitude. Human beings are regarded as “superorganisms” whose metabolism represents an amalgamation of microbial and human attributes (Gill et al., 2006). Recent findings suggest that the microbial community is indispensable for maintaining human health, yet is also capable of eliciting disease. As one of the largest and most complex microbial habitats, the oral cavity harbors hundreds of bacteria that play vital roles in maintaining oral homeostasis and influencing the development of oral diseases, mainly dental caries and periodontal disease (Chen and Jiang, 2014). In addition, oral pathogens may be important players in systemic diseases, such as diabetes (Moodley et al., 2013), infective endocarditis (Beck and Offenbacher, 2005), bacterial pneumonia (Awano et al., 2008), adverse pregnancy outcomes (Han, 2011), inflammatory bowel disease (Ismail et al., 2012), pancreatic cancer (Farrell et al., 2012), and colon cancer (Castellarin et al., 2012). Despite its substantial impact on overall human health, the complexities of the oral bacterial community and how its composition changes when shifting between the healthy and diseased states are far from being fully understood.

Dental caries, otherwise known as tooth decay, is one of the most prevalent chronic infectious diseases, affecting 80–90% of the worldwide population (Selwitz et al., 2007). Individuals are susceptible to this disease throughout their lifetime, and it is the primary cause of tooth loss, oral infection, and pain (Featherstone, 2000). Dental caries is a multifactorial disease that initiates from bacterial shifts within the supragingival dental plaque, a complex biofilm formed on the tooth surfaces, by the aggregation of a group of bacteria in a well-organized manner (Marsh, 2006). A long history of debate is associated with cariogenic theories of dental plaques, which have mainly focused on four hypotheses: the non-specific plaque hypothesis (Schultz-Haudt et al., 1954), the specific plaque hypothesis (Loesche, 1979), the ecological plaque hypothesis (Marsh, 1994), and, recently, the polymicrobial synergy and dysbiosis hypothesis (Hajishengallis and Lamont, 2012).

Traditional cultivation methods have been used to isolate and characterize over 280 oral bacterial species (Aas et al., 2005). *Streptococcus mutans* was the first species isolated from caries lesions and has been considered the main cariogenic bacteria for decades (Clarke, 1924; Loesche et al., 1975). However, a large proportion of oral bacteria cannot be cultivated *in vitro*, which impedes the thorough and in-depth understanding of the natural microbial community residing in dental plaques (Paster et al., 2001). The use of molecular biological techniques, such as denaturing gradient gel electrophoresis (Muyzer et al., 1993), the quantitative real-time polymerase chain reaction (Ciric et al., 2010), microarray chips (Preza et al., 2009), checkerboard hybridization (Wall-Manning et al., 2002), and cloning sequencing of DNA (Kanasi et al., 2010), enables the identification and classification of uncultured oral bacteria. However, these approaches have remarkable biases and drawbacks that do not facilitate the comprehensive study of bacterial diversity, as only the predominant bacteria can be identified while many low-abundance species cannot be detected (Schulze-Schweifing et al., 2014). In recent years, next-generation sequencing technologies have dramatically improved sequencing capabilities (Metzker, 2010) and have been successfully applied for oral microbial analysis in a high-throughput manner (Zaura, 2012; Nyvad et al., 2013; Wade, 2013; Camelo-Castillo et al., 2015), including five major platforms for microbiome studies (namely the 454, Illumina, SOLiD, Ion Torrent, and PacBio platforms) (Hodkinson and Grice, 2015). To date, the overall diversity and structure of supragingival plaque microbiota in adult dental health and caries have not been completely elucidated.

Thus, we used amplicon pyrosequencing of the 16S rDNA V1–V3 hypervariable regions to comprehensively define the bacterial composition, abundance, and structure of supragingival plaques in a relatively large number of adult subjects with dental health and caries. Unlike previous studies, the cases of dental caries were grouped into different categories of severity according to the decayed missing filled tooth (DMFT) index. Furthermore, we aimed to identify bacterial shifts related with dental health and caries, and to investigate the presence of a core plaque microbiome. Our findings expand the understanding of bacterial ecology in dental caries and provide directions for prevention and treatment strategies of dental caries.

## Materials and methods

### Subjects

This study was performed in accordance with the recommendations of the Ethics Committee of the Ninth People's Hospital of Shanghai Jiao Tong University. All subjects gave written informed consent in accordance with the Declaration of Helsinki.

A total of 160 subjects were recruited for this study, including 131 patients with dental caries from the Department of Endodontics (Ninth People's Hospital, Shanghai Jiao Tong University School of Medicine) and 29 dental healthy volunteers from the College of Stomatology (Shanghai Jiao Tong University). Inclusion criteria of subjects were as follows: (Gill et al., 2006) an age of 20–50 years old (male or female), (Chen and Jiang, 2014) good oral hygiene with no bad eating habits, (Moodley et al., 2013) except for dental caries, no other oral diseases (such as periodontal disease, periapical disease, oral mucosal disease, or severe halitosis), (Beck and Offenbacher, 2005) no incidence of antibiotics use within the 3 months prior to sampling, and (Awano et al., 2008) written informed consent. Exclusion criteria were as follows: (Gill et al., 2006) pregnancy or breastfeeding, (Chen and Jiang, 2014) suffering from systemic diseases (such as cardiopathy, diabetes, or hypertension), (Moodley et al., 2013) a long history of medication, and (Beck and Offenbacher, 2005) not providing written, informed consent to participate in this study. The detailed clinical parameters of the 160 participants are shown in Table S1.

The dental health status of each subject was assessed by a professional dentist according to the criteria from the World Health Organization 4th-edition publication of “Oral Health Surveys, Basic Methods.” Moreover, the DMFT index was recorded to measure the severity of each subject's dental caries. In accordance with the DMFT index, the 160 subjects were characterized into four groups:

(1) No-caries (NC; DMFT = 0, *n* = 29)
(2) Low-caries (LC; DMFT ≤ 4, *n* = 32)
(3) Moderate-caries (MC; 4 < DMFT < 8, *n* = 37)
(4) High-caries (HC; DMFT ≥ 8, *n* = 62).

### Sample collection

Sampling was performed 2 h after eating in the morning. Supragingival plaque samples were collected from each subject according to the Manual of Procedures for Human Microbiome Project (http://hmpdacc.org/resources/tools_protocols.php), with minor modifications. Briefly, the sampling site was isolated with cotton rolls and dried with a gentle air stream to avoid saliva contamination. Then, a sterile Gracey curette was used to collect a pooled supragingival plaque from the buccal surface of the maxillary first molar or, if necessary, from its adjacent teeth or contralateral teeth, and to avoid gingival bleeding. Each pooled plaque sample was immediately released from the curette by agitation into a sterile, labeled Eppendorf tube containing 1 mL thioglycollate medium, which was placed on ice. Within 4 h of collection, all samples were transported on ice to our laboratory for DNA extraction.

### DNA extraction

Total bacterial genomic DNA was extracted from all samples using the TIANamp Bacterial DNA Kit (TIANGEN, Beijing, China), following the manufacturer's instructions, and stored at −40°C prior to further analysis. The quantity and quality of extracted DNAs were measured using a NanoDrop ND-1000 spectrophotometer (Thermo Fisher Scientific, Waltham, MA, USA) and agarose gel electrophoresis, respectively. The results showed that the A260:A280 ratios were 1.8–2.0 and that the DNA concentrations were 20–150 ng/μL, indicating that the genomic DNA extracted was ideal and met the requirements for subsequent sequencing.

### 16S rDNA amplicon pyrosequencing

PCR amplification of the bacterial 16S rDNA V1–V3 region was performed using the forward primer 8F (5′-AGAGTTTGATCCTGGCTCAG-3′) and the reverse primer 533R (5′-TTACCGCGGCTGCTGGCAC-3′) (Moreno et al., 2002). Unique 7-bp barcodes were incorporated into the primers for multiplex sequencing. Details of the barcodes used are shown in Table S2. Twenty-five microliter reactions were prepared containing 5U DNA polymerase (Pyrobest, Takara, Japan), 10x Pyrobest buffer II, 2.5 mM dNTPs, 10 μM forward and reverse primers, and 40 ng template DNA. PCR amplification were performed on an ABI 9600 instrument with an initial denaturation at 94°C for 4 min; followed by 25 cycles of denaturation at 94°C for 45 s, annealing at 60°C for 45 s, extension at 72°C for 45 s; and a final extension at 72°C for 8 min. PCR amplicons were purified with Agencourt AMPure Beads (Beckman Coulter, Indianapolis, IN) and quantified using the PicoGreen dsDNA Assay Kit (Invitrogen, Carlsbad, CA, USA). Equimolar concentrations of purified amplicons were pooled together into a single amplicon library, which was then amplified by emulsion PCR according to the manufacturer's instructions. Subsequently, pyrosequencing was performed on the Roche 454 GS FLX+ Titanium platform (Roche, USA), following the vendor's standard protocols.

### Sequence analysis

The Quantitative Insights Into Microbial Ecology (QIIME) pipeline was employed to process the pyrosequencing data, as previously described (Caporaso et al., 2010). Briefly, raw sequences with exact matches to the barcodes were assigned to respective samples and identified as valid sequences whose primers and barcodes were trimmed for further quality control. The filtration criteria of the low-quality sequences were as follows: (Gill et al., 2006) sequences that had a length of <200 bp, (Chen and Jiang, 2014) sequences that had average Phred scores of <25, (Moodley et al., 2013) sequences that had ambiguous bases, and (Beck and Offenbacher, 2005) sequences that had mononucleotide repeats of >6. The remaining high-quality sequences were clustered into operational taxonomic units (OTUs) at 97% sequence identity. A representative sequence from each OTU was assigned taxonomically by BLAST searching against the Human Oral Microbiome Database (HOMD) using the best hit (Altschul et al., 1990). The BLAST parameters used were at least 97% sequence identity >200 bp, as cutoff values. Next, an original OTU table was created that contained a readable matrix of the OTU abundance for each sample and the taxonomic classification for each OTU. Finally, to minimize the effects of random sequencing errors, the original OTU table was modified by removing OTUs containing less than 10 sequences. The modified OTU table was used as a basis for subsequent analysis.

### Bacterial community characterization, statistical analysis, and visualization

In QIIME (version 1.8.0), alpha diversity indexes, including the Chao1, ACE, Shannon, Inverse Simpson, Good's coverage, and Simpson's evenness indexes were calculated at 97% identity (Paul et al., 2016). Student's *t*-test was used to identify significant differences in the α diversity indexes between the different groups (*p* < 0.05) (Signori et al., 2014). A ranked abundance curve was plotted to explain both the richness and evenness of species. Beta diversity was analyzed to investigate the similarity of bacterial community structure among groups using UniFrac distances and visualized via principal coordinate analysis (PCoA) and unweighted pair-group method with arithmetic means (UPGMA) hierarchical clustering analysis (Lozupone et al., 2007). Based on the genus level classification, a principal component analysis (PCA) was also conducted to evaluate the similarity among various bacterial communities (Ramette, 2007). Differences in the Unifrac distances for pairwise comparisons among groups were determined using Student's *t*-test and the Monte Carlo permutation test with 1000 permutations, and visualized by constructing a box-and-whiskers plot. The significance of group-related sample aggregation was assessed by analysis of similarity (ANOSIM) test. The taxonomy compositions and abundances were visualized using MEGAN (version 4.70.4) and GraPhlAn software (version 0.9.7) (Huson et al., 2011; Asnicar et al., 2015). Using mothur software (version 1.31.2), a Venn diagram was generated to show the shared and unique OTUs among groups, based on the occurrence of OTUs in a sample group regardless their relative abundance (Zaura et al., 2009). Shared taxa present in all four groups (100% core threshold) were defined as the core microbiome.

Using QIIME, the taxa abundances at the phylum, class, order, family, genus and species levels were statistically analyzed and plotted to show the distributions among groups. Furthermore, LEfSe (version 1.0) was used to detect differentially abundant genera in the four groups for biomarker discovery using the online Galaxy workflow framework (http://huttenhower.sph.harvard.edu/galaxy/) (Segata et al., 2011); the threshold on the logarithmic linear discriminant analysis (LDA) score for discriminative features was set to 2.0. To identify key genera that were responsible for the differential distributions of plaque microbiota between groups, a partial least squares discriminant analysis (PLS-DA) model was generated using the plsda function of the mixOmics package (version 6.0.0) in R software (version 3.2.0) (Chen et al., 2011). The key genera with variable importance in projection (VIP) > 1 were considered to be important contributors to the model. Using mothur software, co-occurrence analysis among genera was investigated by calculating C-scores, and Spearman's rank correlations of the 50 most abundant genera were calculated. Network analysis using the genera with rho > 0.6 and *p* < 0.01 was visualized using Cytoscape (version 3.4.0) (Shannon et al., 2003). Microbial functions were predicted using PICRUSt (version 1.0.0) and aligned to the Kyoto Encyclopedia of Genes and Genomes (KEGG) database (Langille et al., 2013).

### Data access

All raw sequences were deposited in the NCBI Sequence Read Archive under accession number SRP076428.

## Results

### Global sequencing data

A total of 2,568,368 valid sequences were generated from 160 supragingival dental plague samples, with an average of 16,052 sequences per sample (ranging from 10,281 to 29,718). After data trimming and quality filtering, 2,261,700 high-quality sequences (representing ~88% of the total sequences) were acquired, with an average of 14,136 sequences per sample (ranging from 6631 to 27,428; Table S3). The average sequence length was 488 bp, with the maximum length being 506 bp and the shortest length being 294 bp (Figure S1). Clustering of all high-quality sequences at 97% identity resulted in 58,778 OTUs, which were BLAST-searched against the HOMD database for taxonomic assignments. After removing the low-credibility OTUs (together contributing only 5.3% of all sequences), a modified OTU table was obtained consisting of 10,365 OTUs with an average of 1156 OTUs per sample (ranging from 339 to 2559; Table S4).

### Bacterial diversity analysis

The alpha diversity indices of Chao1, ACE, Shannon, Inverse Simpson, Good's coverage, and Simpson's evenness are shown in Table 1. The Shannon diversity index was higher in NC group than in HC, MC, or LC group, but there was no significant difference between groups by *t*-test. However, the ACE richness index was significantly different between groups NC and HC (993.26 vs. 755.82, *p* = 0.01), the Inverse Simpson diversity index was significantly different between groups MC and HC (29.54 vs. 24.03, *p* = 0.04), demonstrating the higher bacterial diversity of healthy dental plaques compared to dental caries, with the diversity decreasing gradually with the severity of caries. Good's coverage estimator for each group was over 95%, indicating that the current sequencing depth was sufficient to saturate the bacterial diversity of dental plaques. In addition, Simpson's evenness index indicated that the bacterial-community distribution in the plaque samples was very uneven, which was also observed in the rank-abundance curve which had a high slope and a long tail comprised of low-abundance OTUs (Figure S2). The 200 most abundant OTUs represented 75.4% of all sequences. Most of the remaining OTUs were present at low abundance.

**Table 1:** **Alpha diversity indices for supragingival plaque bacteria in each group at 97% identity**.

**Group**	**Chao1**		**ACE**		**Shannon**		**Inverse Simpson**		**Coverage**		**Simpsoneven**	
	**Mean**	**SE**	**Mean**	**SE**	**Mean**	**SE**	**Mean**	**SE**	**Mean**	**SE**	**Mean**	**SE**
HC	464.71	26.53	755.82^*^	50.59	6.03	0.13	24.03^*^	1.79	0.96	0.00	0.03	0.00
LC	510.72	37.19	847.11	72.24	6.17	0.19	27.19	3.31	0.96	0.00	0.03	0.00
MC	482.30	34.84	792.19	70.51	6.26	0.15	29.54^*^	2.74	0.96	0.00	0.03	0.00
NC	551.48	34.20	993.26^*^	75.64	6.37	0.17	28.62	2.64	0.95	0.00	0.02	0.00

SE, Standard Error. Asterisk indicates significant differences (p < 0.05, Student's t-test).

* ACE index between NC and HC was statistically significant different (p = 0.01).

* Inverse Simpson index between HC and MC was statistically significant different (p = 0.04).

### Bacterial abundance and distribution

**Figure 1:**
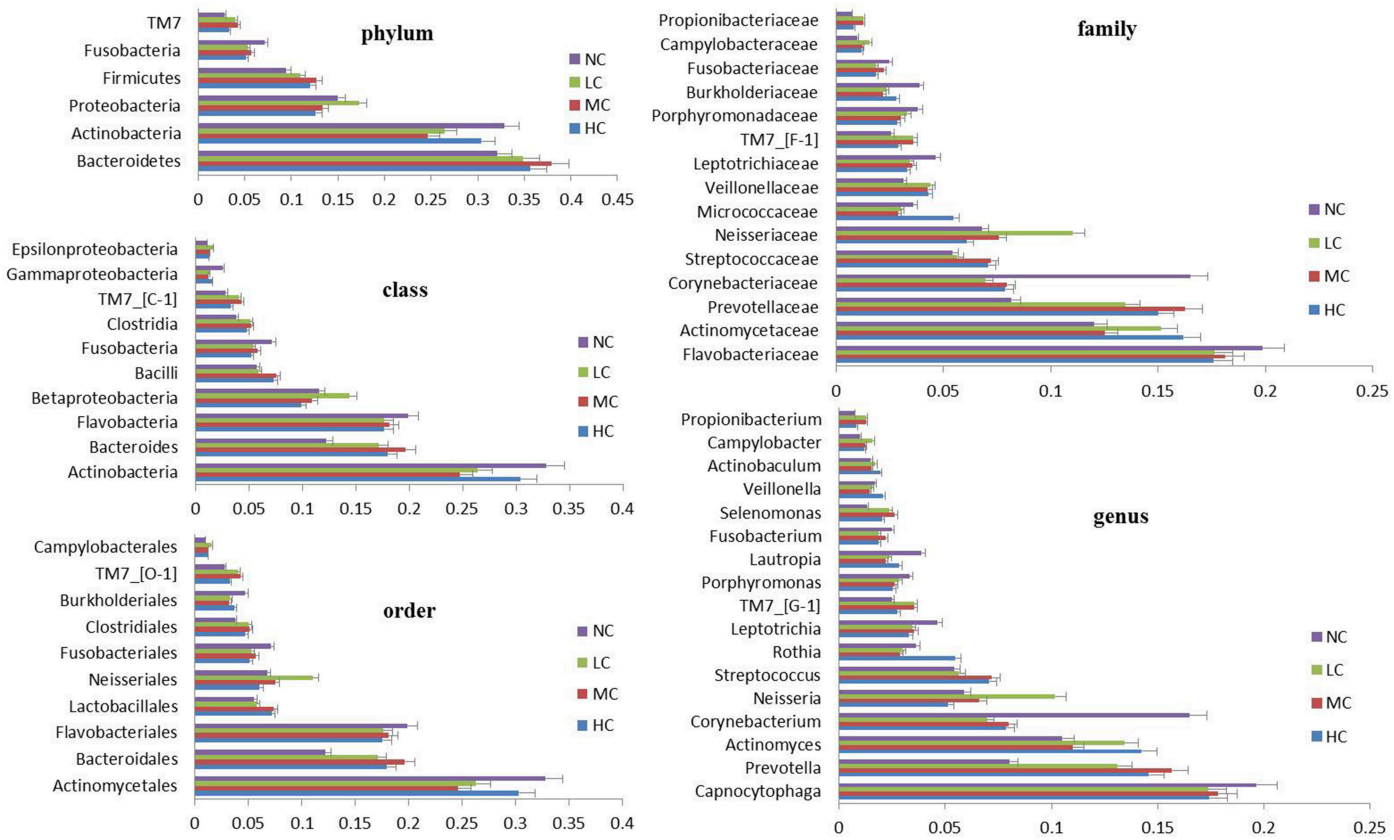
**Distribution of the predominant bacteria at different taxonomic levels (phylum, class, order, family, and genus)**. The predominant taxa (>1% relative abundance) in each level are shown.

**Figure 2:**
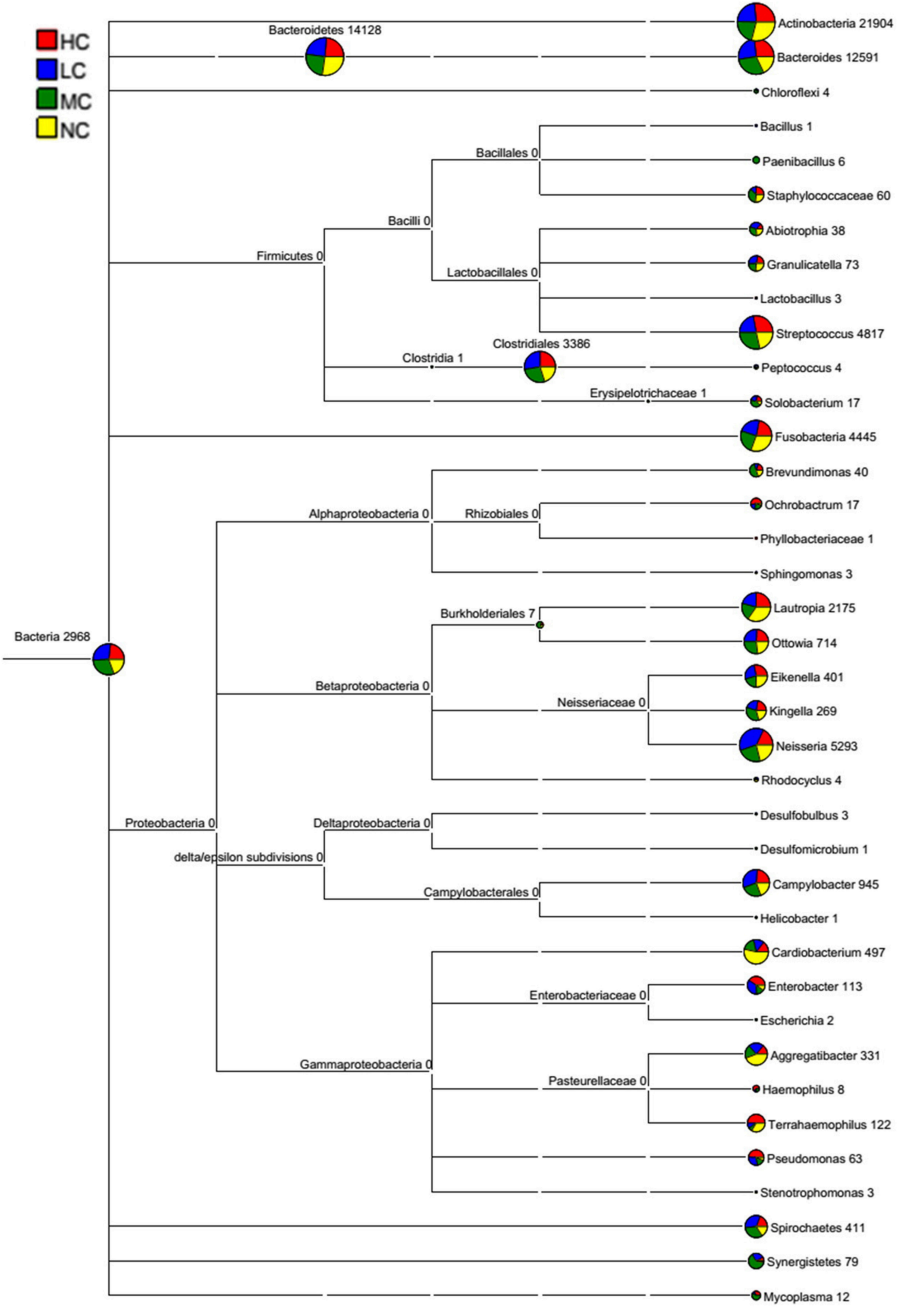
**A classification tree showing bacterial abundance by MEGAN**. The taxonomy compositions and abundances were visualized by MEGAN (version 4.70.4). The larger the area of the colored pie chart, the greater the bacterial abundance. Different colors represent different groups, and the larger the colored sectorial area at a branch, the more the corresponding group contributed to the bacterial abundance.

**Figure 3:**
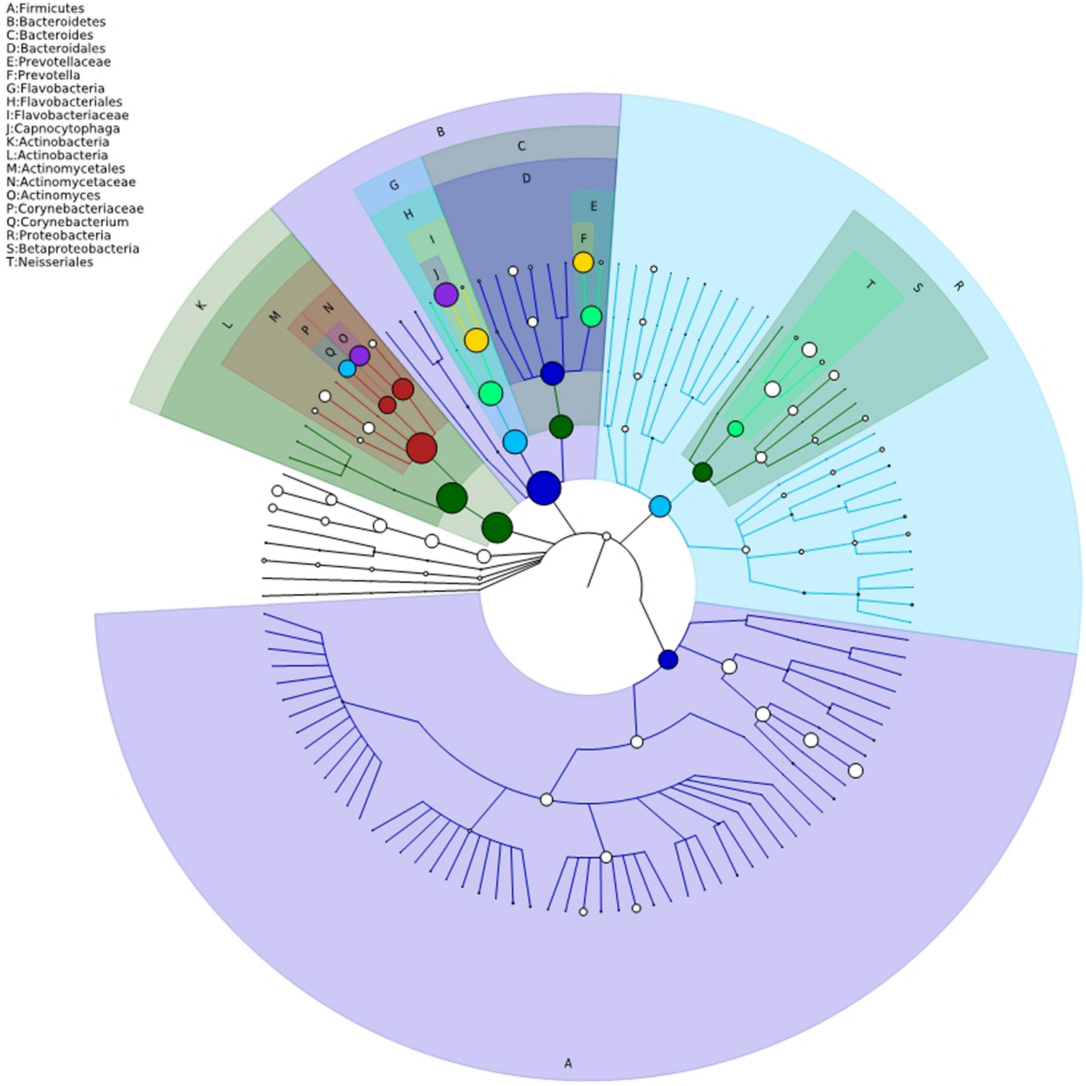
**Taxonomy visualization**. A taxonomy tree visualized by GraPhlAn. The background color of the letter is consistent with the color of corresponding node. The colored nodes from the inner to the outer circles represent the top 20 abundant taxa from the phylum to genus level, which are signified by the letters arranged from the outer to the inner circles.

The bacterial distribution was characterized in terms of the relative taxonomic abundances. A total of 12 phyla, 21 classes, 34 orders, 66 families, 122 genera and 453 species were detected in the supragingival plaque samples. Figure 1 shows taxonomic distributions of the predominant bacteria (relative abundance >1% of the total sequences) at different levels. The 6 most abundant phyla were *Bacteroidetes* (35.1% of the total sequences), *Actinobacteria* (28.6%), *Proteobacteria* (14.6%), *Firmicutes* (11.3%), *Fusobacteria* (5.8%), and *TM7* (3.6%), together accounting for 99% of the total sequences. The 6 most rare phyla were *Spirochaetes, SR1, Synergistetes, GN02, Tenericutes*, and *Chloroflexi*. The most prevalent genera were *Capnocytophaga* (18.1%), *Prevotella* (12.8%), *Actinomyces* (12.3%), *Corynebacterium* (9.8%), *Neisseria* (7.0%), *Streptococcus* (6.3%), *Rothia* (3.7%), *Leptotrichia* (3.7%), *TM7_[G-1]* (3.1%), *Porphyromonas* (2.8%), *Lautropia*, (2.8%), *Fusobacterium* (2.1%), *Selenomonas* (2.1%), *Veillonella* (1.7%), *Actinobaculum* (1.7%), *Campylobacter* (1.2%), and *Propionibacterium* (1.0%), together comprising 92.3% of the total sequences. The compositions in taxa of the microbial communities according to the tested sample groupings are provided in Figure 2. The 7 genera *Neisseria, Streptococcus, Lautropia, Campylobacter, Ottowia, Cardiobacterium*, and *Aggregatibacter* were relatively abundant, with higher abundances of *Lautropia, Cardiobacterium*, and *Aggregatibacter* observed in the NC group and higher abundances of *Neisseria* and *Campylobacter* observed in the LC group. Nevertheless, the *Streptococcus* and *Ottowia* genera exhibited similar abundances in each group. These significant differences were further tested by LEfSe and PLS-DA analysis. A taxonomy tree visualized by GraPhlAn was used to quickly find the dominant taxa from the complex microbial data. As shown in Figure 3, the colored nodes represented the top 20 most abundant taxa signified by the letters in the tree, including *Firmicutes, Bacteroidetes* (*Prevotella, Capnocytophaga*), *Actinobacteria* (*Actinomyces, Corynebacterium*) and *Proteobacteria*.

### Bacterial community structure

**Figure 4:**
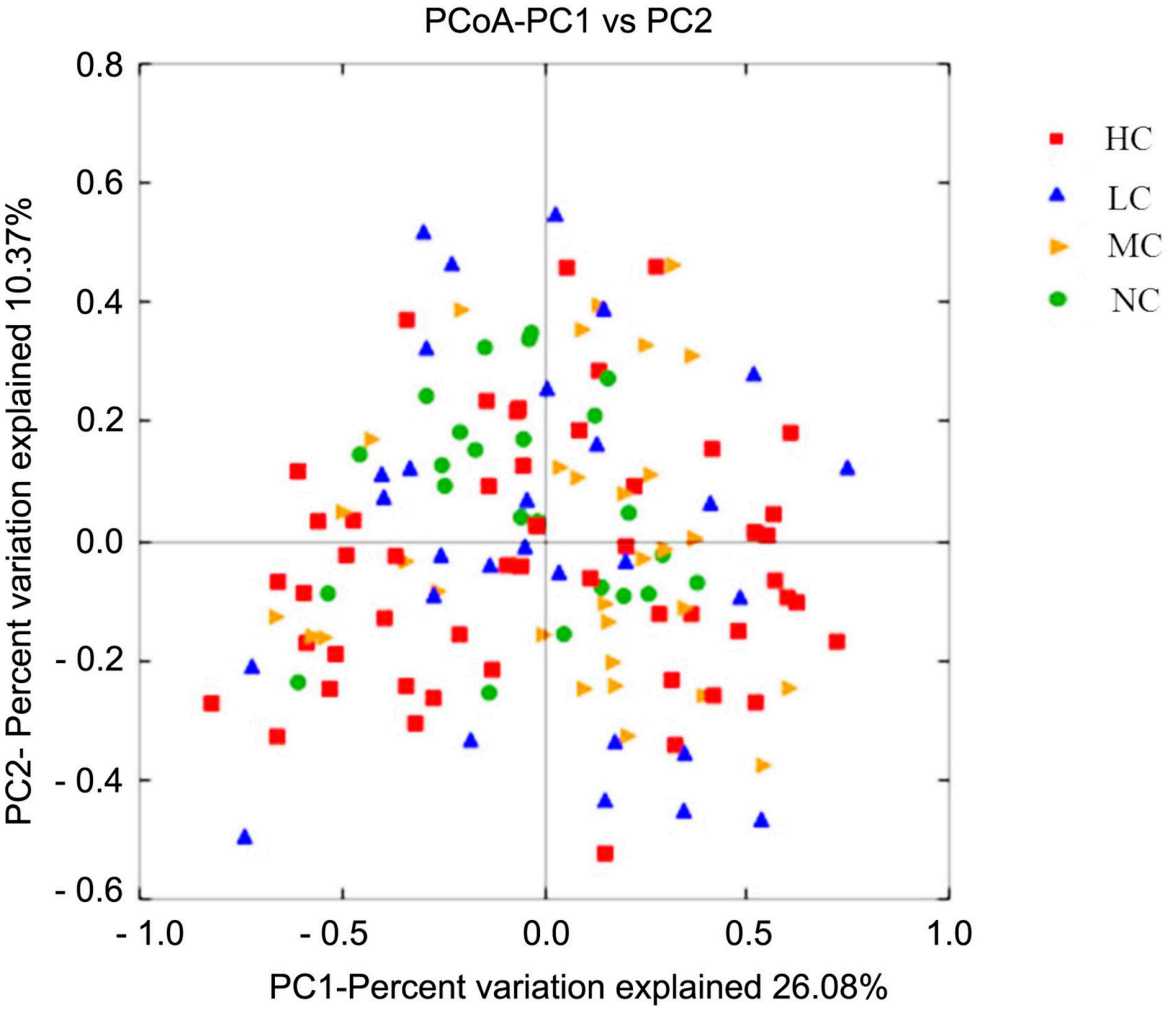
**PCoA based on the weighted UniFrac distances at the OTU level at 97% identity**. Each sample is represented by a dot. Red squares represent the HC samples. Orange triangles represent the MC samples. Blue triangles represent the LC samples. Green balls represent the NC samples. PC1 explained 26.08% of the variation observed, and PC2 explained 10.37% of the variation. However, the samples did not form well-separated clusters corresponding to the four groups, suggesting that the bacterial structures in healthy and caries groups were similar.

To gain insight into similarities in the bacterial community structures among the four groups, PCoA of beta diversity analysis was performed based on the weighted UniFrac distances, which demonstrated similar community structures among healthy and caries groups. As shown in Figure 4, NC microbiota overlapped with some HC, MC, and LC microbiota. Furthermore, the results of PCA based on the genus level classification exhibited no clear segregations in community structures among groups, with the first 2 principal components representing 28.39% and 24.1% of the total variations (Figure S3). ANOSIM testing confirmed that no significant separation occurred among groups at the level of *P* = 0.462. UPGMA hierarchical clustering analysis also revealed that the samples did not form well-separated clusters corresponding to the four groups, indicating similarity in the bacterial community structures (Figure S4). Moreover, differences in Unifrac distances for pairwise comparison among groups were found (Figure S5).

### Differential microbiota compositions

**Figure 5:**
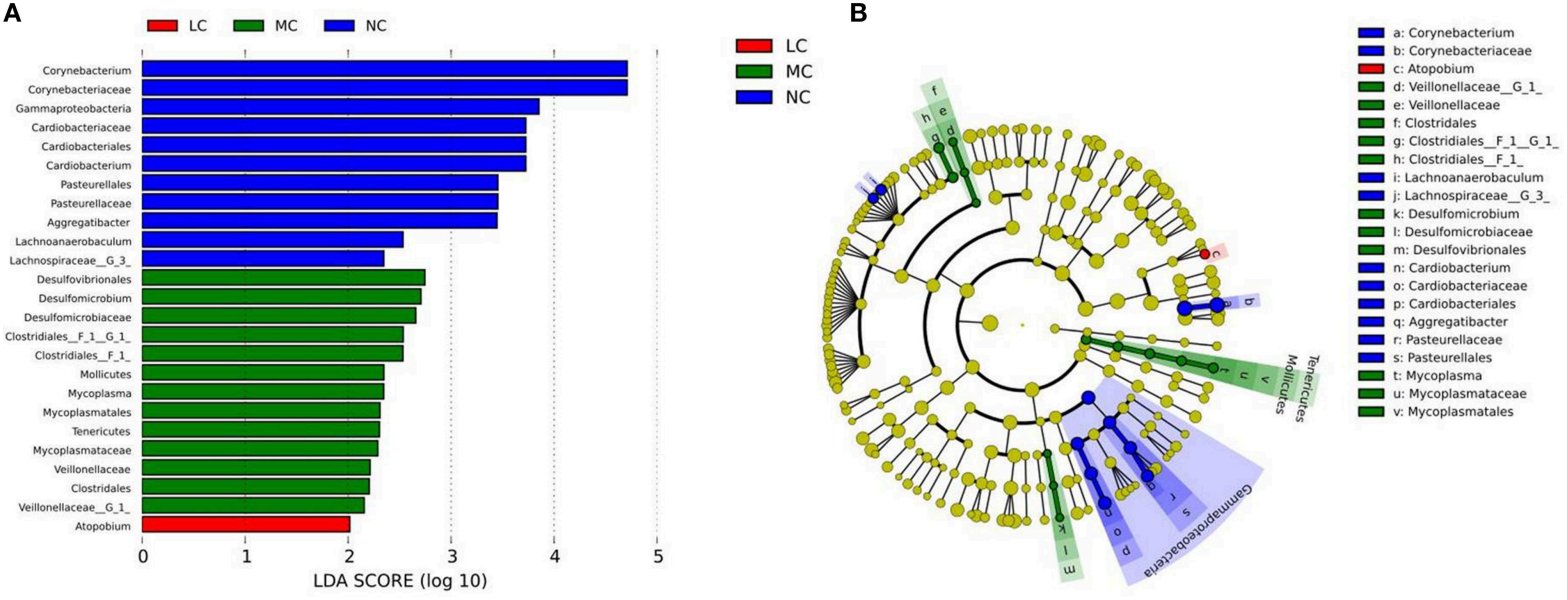
**Comparison of microbial variations at the genus level, using the LEfSe online tool. (A)** Histogram of the LDA scores for differentially abundant features among groups. The threshold on the logarithmic LDA score for discriminative features was set to 2.0. **(B)** Cladogram for taxonomic representation of significantly differences among groups. Differences are represented in the color of the most abundant taxa (red indicating LC, green indicating MC, blue indicating NC, and yellow indicating non-significant).

There were significant differences in the community compositions among the four groups. As shown in Figure 5, a cladogram representation of significantly different taxa among groups was performed by LEfSe. *Tenericutes* was significantly enriched in MC group. There were 7 significantly different families, with the enrichment of *Desulfomicrobiaceae, Mycoplasmataceae, Clostridiales__F_1* and *Veillonellaceae* in MC group, *Corynebacteriaceae, Pasteurellaceae*, and *Cardiobacteriaceae* in NC group. The microbial composition was also significantly different at the genus level, with 10 significantly different genera among groups. *Aggregatibacter, Lachnoanaerobaculum, Corynebacterium, Lachnospiraceae_G_3*, and *Cardiobacterium* exhibited a relatively higher abundance in NC group. *Desulfomicrobium, Mycoplasma, Clostridiales_F_1_G_1*, and *Veillonellaceae_G_1* were relatively more abundant in MC group. *Atopobium* was significantly enriched in LC group. These differentially abundant taxa can be considered as potential biomarkers (LDA > 2, *p* < 0.05). Since LEfSe was a strict tool, we also generated a PLS-DA model to identify more genera that were differentially distributed among groups. The key genera with VIP > 1 were considered to be important contributors to the model. As shown in Table 2, a total of 27 genera with a VIP score > 1 were identified as key genera responsible for significant differences in the community composition. Among them, 10 genera were significantly enriched in NC group, including *Cardiobacterium, Corynebacterium, Lachnospiraceae_[G-3], Lachnoanaerobaculum, Aggregatibacter, Eubacterium_[XI][G-7*], *GN02_[G-1], Fusobacterium, Clostridiales_[F-2][G-1]*, and *Tannerella*, which were identified as health-related bacteria. The other 17 genera were significantly more abundant in HC, MC and LC groups, identified as caries-related bacteria. Furthermore, two genera were identified with a VIP > 2, namely the *Cardiobacterium* and *Corynebacterium* genera.

**Table 2:** **Key genera responsible for differential distributions according to the PLS-DA model**.

**Genus**	**VIP Score**	**Enriched group**
*Cardiobacterium*	2.63	NC
*Corynebacterium*	2.52	NC
*Lachnospiraceae_[G-3]*	1.64	NC
*Lachnoanaerobaculum*	1.44	NC
*Aggregatibacter*	1.34	NC
*Eubacterium_[XI][G-7]*	1.30	NC
*GN02_[G-1]*	1.12	NC
*Fusobacterium*	1.38	NC
*Clostridiales_[F-2][G-1]*	1.36	NC
*Tannerella*	1.22	NC
*Veillonella*	1.41	HC
*Eikenella*	1.34	HC
*SR1_[G-1]*	1.22	HC
*Lachnospiraceae_[G-8]*	1.07	MC
*Flavobacteriales_[G-1]*	1.03	MC
*Clostridiales_[F-1][G-1]*	1.02	MC
*Catonella*	1.16	MC
*Peptostreptococcus*	1.02	MC
*Prevotella*	1.02	MC
*Selenomonas*	1.01	MC
*Fretibacterium*	1.72	MC
*Bacteroidetes_[G-5]*	1.26	LC
*Campylobacter*	1.11	LC
*TM7_[G-3]*	1.32	LC
*Leptotrichia*	1.28	NC
*Bacteroidales_[G-2]*	1.20	LC
*Dialister*	1.10	LC

A total of 27 genera with VIP > 1 were identified as key genera responsible for differential distributions.

### Core microbiome

**Figure 6:**
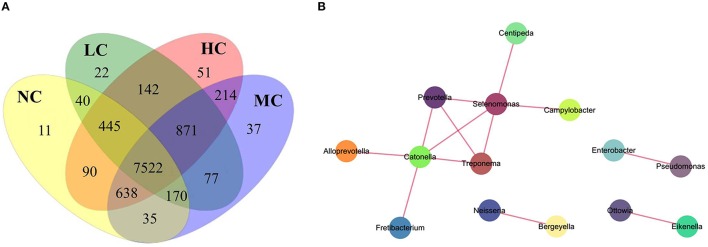
**A Venn diagram and network analysis. (A)** A Venn diagram showing shared and unique OTUs at 97% identity among the four groups. **(B)** A network diagram of dominant genera showing positive associations.

To examine the existence of an identifiable common core microbiome (Lozupone et al., 2007), we defined a core as the group of members shared among the microbial community and represented the core by overlapping areas in the circles in a Venn diagram, at 97% identity. We identified 8951, 9289, 9564, and 9.973 OTUs in the NC, LC, MC, and HC groups, respectively. As shown in Figure 6A, 7522 OTUs were shared among the four groups, occupying 72.6% of all OTUs (10,365 OTUs) and 97.5% of all OTU abundances. Furthermore, 12 shared (100% of total numbers) phyla, 20 shared (95.2%) classes, 30 shared (88.2%) orders, 54 shared families (81.8%), and 99 shared genera (81.1%) were identified. These shared taxonomic members can be regarded as the core microbiome of supragingival plaques. Among the 99 core genera, the 6 most abundant genera were *Capnocytophaga* (17.8% of total abundances), *Prevotella* (13.5%), *Actinomyces* (13.0%), *Corynebacterium* (8.9%), *Streptococcus* (6.6%), and *Neisseria* (6.4%).

In the Venn diagram, the unique OTUs in each group were also observed with 11, 22, 37, and 51 unique OTUs found in the NC, LC, MC, and HC groups, respectively. These unique OTUs were in low abundance, only containing 10–297 sequences, and can be considered as a variable microbiome. The 11 unique OTUs in the NC group belonged to 9 species: *Propionibacterium* sp.*_oral_taxon_915, Propionibacterium propionicum, Capnocytophaga* sp.*_oral_taxon_326, Bergeyella* sp.*_oral_taxon_900, Porphyromonas* sp.*_oral_taxon_279, Selenomonas infelix, Dialister invisus, Cardiobacterium hominis*, and *Neisseria sicca*, which were identified as health-specific species. The unique OTUs in the LC, MC, and HC groups are shown in Table S5.

### Co-occurrence network analysis and function predictions

Co-occurrence analysis was used to discern relationships among the plaque microbiota at the genus level by calculating C-scores. As shown in Table 3, the *p*-value was > 0.95 in all samples, suggesting that the plaque microbiota displayed a symbiotic relationship, i.e., a co-occurrence pattern. As shown in the network diagram for the 50 most abundant genera (Figure 6B), 14 genera displayed positive associations. Among them, *Selenomonas* and *Catonella* exhibited a high degree of linkages with other genera. High rho values were found for the Pseudomonas–Enterobacter (0.81), Selenomonas–Prevotella (0.76), and Treponema–Prevotella (0.75) pairs.

**Table 3:** **Co-occurrence analysis by calculating C-scores**.

		**Average C-score**	***P*-value**
Group	all	0.483	1
	HC-LC	0.501	0.663
	HC-MC	0.498	0.997
	HC-NC	0.499	0.992
	LC-NC	0.499	0.997
	LC-MC	0.500	0.956
	MC-NC	0.496	1
Sample	all	0.490	1

p-value > 0.95 suggests that the microbiota displayed a co-occurrence relationship.

To predict bacterial functions of members of the supragingival plaque community, PICRUSt analysis was performed based on the 16S rRNA composition data of each sample (Figure S6). A total of 41 metabolic functions were predicted in all samples with the most enrichment in membrane transport (10.0%), carbohydrate metabolism (9.3%), replication and repair (9.1%), amino acid metabolism (9.0%), translation (6.3%), and energy metabolism (5.6%). We also investigated bacterial functions associated with human diseases such as infection diseases, cancers, neurodegenerative diseases, cardiovascular diseases, metabolic diseases, and immune system diseases.

## Discussion

A comprehensive and thorough investigation of the bacterial diversity of plaque microbiota is essential for understanding their etiologies and for developing effective prevention and treatment strategies of dental caries. The introduction of high-throughput pyrosequencing has provided new insights into the compositions and structures of microbial communities. We used 454 FLX+ pyrosequencing to explore the bacterial diversity and community structure of 160 supragingival plaque samples by sequencing the 16S rDNA hypervariable V1–V3 region in samples from 131 adults with dental caries and 29 volunteer with good oral health. Different from previous studies using basic molecular techniques such as DGGE, qPCR and microarray chips (Muyzer et al., 1993; Preza et al., 2009), we not only identified the predominant taxa but also a very large number of low-abundance or rare taxa such as *Ochrobactrum, Centipeda, Lachnoanaerobaculum, GN02_[G-1], Moryella, Bacteroides, Megasphaera, Rhizobium, Filifactor*, and *Dietzia*. The V1–V3 region has been used in several studies (Kumar et al., 2011; Xu et al., 2014; Yoo et al., 2016) because it can provide greater phylogenetic resolution and better analysis of diversity and richness. In addition, this region has more sequences deposited into databases, which greatly facilitates diversity analysis (Kim et al., 2011).

We obtained 2,261,700 high-quality sequences with an average of 14,136 sequences per sample (Table S3), which was much higher than reported in previous studies (Keijser et al., 2008; Ling et al., 2010). For pyrosequencing studies of 16S rRNA, a depth of coverage of approximately 1000 sequences per sample provided a good balance between sample number and sampling depth, and to enabled detection of species at 1% abundance with reasonable accuracy (Hamady and Knight, 2009). Thus, the sequencing depth in our study (14,136 sequences per sample) was reasonable and large enough to enable detection of the vast majority of bacterial species in plaque samples. Furthermore, a Good's coverage estimator of >95% also suggested that our sequencing depth was sufficient to reflect the full bacterial diversity of plaque microbiota (Table 1), indicating that some extremely low abundance of rare species could be detected and might be related to dental caries (Schloss et al., 2011).

At 97% identity, 10,365 OTUs belonging to 453 species, 122 genera, 66 families, 34 orders, 21 classes, and 12 phyla were obtained after filtering out the low-credibility OTUs and BLAST searching against the HOMD database for the taxonomic assignment. In agreement with the results of the present study, many investigators have used 97% identity to classify microorganisms at the species level (Acinas et al., 2004; Yarza et al., 2008; De Gannes et al., 2013; Lindeque et al., 2013). Other studies have explored the classification of oral microbiota. For instance, Pushalkar et al. (2011) identified 860 species, 52 genera, and 8 phyla; Ling et al. (2010) identified 203 genera and 10 phyla; Keijser et al. (2008) identified 318 genera and 22 phyla; and Siqueira and Rôças (2009) identified 460 species, 100 genera, and 9 phyla. The differences in microbial taxonomy observed among these studies may be due to differences related to the selection process for subjects, the geographic location, oral sample collection sites, target sequencing regions of 16S rDNA, databases, and the data-analysis methods.

Compared with Xu's study of supragingival plaque microbiota diversity in children younger than 30 months ranging from 125 to 255 OTUs per sample (Xu et al., 2014), we observed a high diversity in adults aging 20–50 years with dental health or caries ranging from 339 to 2559 OTUs per sample. This suggested that the diversity of oral microbiota increased with age and developmental status, such as primary and permanent tooth growth (Papaioannou et al., 2009). Tsai et al recently identified an average of 774 classified phylotypes per sample and a total of 6 phyla across all subgingival samples in individuals with severe chronic periodontitis (Tsai et al., 2016). In contrast, our study found high diversity with an average of 1156 OTUs per sample and 12 phyla across all supragingival samples in adults with dental caries. We have confirmed that sites in the oral cavity had distinct bacterial diversities that changed with disease status, as reported previously (Paster et al., 2006).

By comparing the alpha diversity indexes among groups using Student's *t*-test, we found that the ACE richness index in the NC group was significantly higher than in the HC group (*p* = 0.01), indicating that the bacterial diversity of healthy dental plaques was higher than that in caries. This finding was in agreement with results from previous studies (Li et al., 2005; Preza et al., 2008). Considered from a bacterial point of view, a more diverse community represents a more healthy and stable ecosystem. The carbohydrate-driven lowering of the pH from lactate produced by acid-producing species could lead to suppression of acid-sensitive species and overgrowth of acid-tolerant species, resulting in decreased bacterial diversity in supragingival plaques as caries progressed, as well as a decreasing number of species capable of surviving harsh conditions (Gross et al., 2010). Li et al. also reported that the species richness and Shannon diversity index were greater in caries-free children than in those with severe early childhood caries (Li et al., 2007). Our study provided additional support for the “ecological catastrophe” of dental caries occurring between commensal microbiota and the host, confirming a decline of bacterial diversity as the severity of caries increased (Marsh, 2003). Some bacteria in dental caries might become lost, inhibited, or replaced, although these possibilities require further study to establish their validity.

The existence of a “core microbiome” was first proposed by Turnbaugh et al. (2007) and referred to the organisms, genes, or functions shared by all or most individuals in a given human habitat, such as the oral cavity, nasal cavity, skin, and intestinal tract. One goal of the Human Microbiome Project was to determine whether an identifiable core microbiome exists among individuals. Most studies conducted to date have focused on the human intestinal tract and investigated the relationships between the core gut microbiome and diseases (e.g., obesity, diabetes, and metabolic syndrome) (Tschöp et al., 2009; Turnbaugh and Gordon, 2009; Neu et al., 2010; Tilg, 2010). Zaura et al. provided the first insight into the oral core microbiome using 454 pyrosequencing technology and found 387 overlapping OTUs in 3 healthy individuals (Zaura et al., 2009). In our study, 7522 overlapping OTUs were shared by the four groups in a Venn diagram (Figure 6A), defined as plaque core microbiome, regardless of whether it had high or low abundance. These widespread core microbiomes might play important roles in the stability and functions of the plaque microbiota. By PICRUSt analysis, we predicted bacterial functions mainly enrichment in membrane transport, carbohydrate metabolism, replication and repair, amino acid metabolism, translation, and energy metabolism. As suggested by Shade (Shade and Handelsman, 2012), beginning with a Venn diagram representing membership, layers of complexity are added by incorporating the composition, phylogeny, persistence, and connectivity to define core microbiomes. The appropriate definition of a core microbiome depends on the ecological question addressed. Our study mainly aimed to determine the presence of a core microbiome, so we typically defined a core as the group of members shared among the microbial community and represented it by the overlapping areas of circles in Venn diagrams. In a future study, we will apply multiple definitions to the core microbiome to enhance the ecological meaning.

Our results indicated that similar community structures of supragingival plaques were present in the four groups according to PCoA and hierarchical clustering analysis (Figure 4 and Figure S4). Samples from the healthy group tended to cluster together, whereas the caries microbiota appeared to be more variable, which was consistent with the results of Yang's study (Yang et al., 2012). The bacteria composing each group were relatively constant. It can be inferred that bacterial components of dental plaques were not markedly affected by the disease status or pathological factors. In spite of the presence of similar bacterial members, the abundances of some bacteria differed significantly among the groups. Twelve bacterial phyla were represented in all supragingival plaque samples. Six predominant phyla were *Bacteroidetes, Actinobacteria, Proteobacteria, Firmicutes, Fusobacteria*, and *TM7*, constituting 99% of the total microbiota (Figure 1). These dominant phyla were largely the same as those previously described for childhood dental caries using pyrosequencing (Jiang et al., 2013).

At the genus level, 122 different genera were identified in the plaque samples of dental health and caries. Seventeen genera had abundances higher than 1% (Figure 1), of which the genera *Actinomyces, Streptococcus, Rothia, Selenomonas*, and *Veillonella* have been detected in early caries studies (Munson et al., 2004; Tanner et al., 2011). The dental plaque biofilm was composed of complex microbial communities, instead of a simple superposition of bacteria. After the formation of acquired pellicles on the teeth, the free bacteria in the oral cavity adhered to pellicles in a definite sequence, thus governing the formation of mature dental plaques (Li et al., 2004). The initial colonizing bacteria was *Streptococcus sanguis*, followed by members of the *Actinomyces, Neisseria, Veillonella, Capnocytophaga, Leptotrichia*, and *Haemophilus* genera, collectively known as early colonizing bacteria. These bacteria can quickly adapt to new environments by regulating specific gene expression and providing new adhesion receptors for later colonizing bacteria, such as members of the *Fusobacterium, Treponema, Tannerella, Prevotella*, and *Porphyromonas* genera (Haffajee et al., 2008). With an increasing number of bacteria in dental plaques, the concentrations of signaling molecules increased, which can activate the expression of related genes, such as virulence factors and mucopolysaccharides. Certain bacteria gradually decrease in abundance, due to the antagonism of predominant bacteria. Consequently, the ecological balance of plaque bacterial microbiota may be broken, eventually leading to the occurrence and development of dental caries (Reading and Sperandio, 2006). We clearly observed that some bacteria in the supragingival plaques of dental caries showed a significantly reduced abundance, such as those in the *Aggregatibacter, Cardiobacterium, Corynebacterium, Lachnoanaerobaculum, Porphyromonas*, and *Tannerella* genera. *Aggregatibacter, Porphyromonas*, and *Tannerella* genera have been associated with dental health (Alcaraz et al., 2012; Jiang et al., 2014). However, the abundance of other bacteria significantly increased, such as *Atopobium, Cryptobacterium, Lactobacillus, Mogibacterium, Ochrobactrum, Pseudomonas, Rhizobium, Alloprevotella, Bacteroides, Centipeda, Campylobacter, Megasphaera*, and *Mycoplasma*; thus, they were regarded as potentially cariogenic bacteria. Some are well-recognized cariogenic bacteria (*Lactobacillus*) or have been identified at significantly higher levels in dental caries (*Cryptobacterium, Atopobium*) (Jiang et al., 2014; Obata et al., 2014). While others may be recognized as new cariogenic bacteria, such as *Catonella, Centipeda, Fretibacterium, Rhizobium, Ochrobactrum*, and *Mogibacterium*. Furthermore, we screened potential biomarkers using LEfSe analysis, including *Desulfomicrobium, Mycoplasma, Corynebacterium, Clostridales, Gammaproteobacteria, Cardiobacterium*, and *Pasteurellaceae*. Therefore, dental caries is a polymicrobial disease caused by various bacterial consortia that are present commensally in healthy individuals at lower levels.

According to our PLS-DA analysis, 27 key genera were identified that were responsible for differential distribution with VIP > 1 (Table 2). Two bacteria with a VIP > 2 (*Cardiobacterium* and *Corynebacterium*) were also identified as health-related genera. *Cardiobacterium* is a member of the group of HACEK bacteria (*Haemophilus, Actinobacillus, Cardiobacterium, Eikenella*, and *Kingella*), which are part of the normal oropharyngeal flora and have common characteristics that easily lead to endocardial infections (Hagiya et al., 2014). Although *Cardiobacterium* bacteria have been reported to relate with aortic valve infective endocarditis and ventricular septal defects (Vanerková et al., 2010; Choudhury et al., 2013), its relationship with oral health and disease has rarely been studied. The *Cardiobacterium* genus has only 2 species, namely *C. hominis* and *Cardiobacterium valvarum*. In our study, *C. hominis* was identified as health-related species that might play an important role in maintaining dental health. Moreover, *Corynebacterium* bacteria were considered as part of a candidate signature for dental health (Peterson et al., 2013). We found that *Corynebacterium matruchotii* was significantly more abundant in dental health than in caries and was identified as a health-related species, which was consistent with the results of Gross's study (Gross et al., 2010).

*S. mutans* was recognized as the main cariogenic bacteria because of its strong capacity for acid production and acid tolerance, as well as its capacity for exopolysaccharide production (Loesche, 1986). Thus, it was surprising in our study that *S. mutans* showed a low abundance in supragingival plaque (only 0.046% in all samples), and showed no significant difference among the groups. In Aas's study, *S. mutans* was not detected in 10% of dental caries using checkerboard hybridization (Aas et al., 2008). Furthermore, Aas et al. found that the proportion of *S. mutans* detected in cavitated dentin lesions was high, whereas the proportion in surfaces of intact enamel and white-spot lesions was very low, which agreed with our results. We speculate that the distribution of *S. mutans* in different oral ecological sites might be heterogeneous and regional. Determining the importance of *S. mutans* in caries development requires a comprehensive consideration of its virulence factors. *Streptococcus sobrinus* was also recognized as cariogenic bacterium. Nevertheless, we observed a low proportion of *S. sobrinus* in the plaque samples. Hirose et al. found that *S. sobrinus* in saliva of children could not only cause pit-fissure caries, but also smooth-surface caries, and suggested that *S. sobrinus* has stronger cariogenic ability than does *S. mutans* (Hirose et al., 1993). It has been demonstrated that *S. sobrinus* is significantly more acidogenic than *S. mutans* and is capable of sustained acid production at pH levels below 6.0 (de Soet et al., 1989; Madison et al., 1991). In our study, *S. sobrinus* was statistically more abundant in caries. Interestingly, we found that *S. sobrinus* was absent in the NC group and was thus regarded as a caries-specific species. Thus, *S. sobrinus* perhaps deserves more attention as cariogenic bacterium.

## Conclusions

In summary, 454 pyrosequencing technology has greatly expanded our knowledge regarding the bacterial diversity and community structure of supragingival plaques in adult dental health and caries. We observed a vast bacterial diversity of supragingival plaques with 2,261,700 high-quality sequences and 10,365 OTUs at 97% identity, which belonged to 453 species, 122 genera, 66 families, 34 orders, 21 classes, and 12 phyla. Bacterial diversity in dental health was higher than in caries and gradually decreased with the severity of dental caries. Our results indicated the occurrence of similar community structures of supragingival plaques among groups. An identifiable core plaque microbiome was determined among the four groups with 7522 overlapping OTUs. Furthermore, we identified a list of caries-related and health-related bacteria, whose specific functions need further testing and verification. Given the polymicrobial and dysbiotic nature of dental caries, diagnostic and preventive strategies directed against specific bacteria would not be universally effective. Regarding preventive and therapeutic approaches, passive immunization strategies are predicted to be inefficient, and the anticipated effectiveness of active immunization strategies, such as caries vaccination, is also uncertain. We believe that the treatment of dental caries should involve balance restoration and that strategies for modulating interactions in the oral microbiota (i.e., molecular interactions, biological microbe-microbe and microbe-host interactions, and microbial ecological interactions), should be further developed.

## Author contributions

Conceived and designed the experiments: JL and ZH. Performed the experiments: CX and SR. Analyzed the data: CX and ZH. Contributed reagents/materials/analysis tools: CX and SR. Manuscript preparation: CX. Manuscript revisions: JL, ZH, CX, and SR.

### Conflict of interest statement

The authors declare that the research was conducted in the absence of any commercial or financial relationships that could be construed as a potential conflict of interest.

## Supplementary material

Table S1 **Clinical parameters of the 160 participants**.

Table S2 **Barcodes (7-base) of all samples subjected to multiplex pyrosequencing**.

Table S3 **Sequences and modified OTUs of all supragingival plaque samples from 454 pyrosequencing at 97% identity**.

Table S4 **Modified OTU table at 97% identity**.

Table S5 **Unique OTUs in the HC, MC, and LC groups**.

Figure S1 **Length distribution of sequences determined by 454 pyrosequencing**.

Figure S2 **Rank abundance curves for all OTUs**.

Figure S3 **Principal component analysis (PCA) at the genus level**.

Figure S4 **UPGMA hierarchical clustering analysis**.

Figure S5 **Box-and-whiskers plot of the Unifrac distance**.

Figure S6 **Bacterial function prediction by PICRUSt analysis**.

## References

[B1] Aas J. A. Griffen A. L. Dardis S. R. Lee A. M. Olsen I. Dewhirst F. E.. (2008). Bacteria of dental caries in primary and permanent teeth in children and young adults. J. Clin. Microbiol. 46, 1407–1417. 10.1128/JCM.01410-07 18216213 PMC2292933

[B2] Aas J. A. Paster B. J. Stokes L. N. Olsen I. Dewhirst F. E. (2005). Defining the normal bacterial flora of the oral cavity. J. Clin. Microbiol. 43, 5721–5732. 10.1128/JCM.43.11.5721-5732.2005 16272510 PMC1287824

[B3] Acinas S. G. Klepac-Ceraj V. Hunt D. E. Pharino C. Ceraj I. Distel D. L.. (2004). Fine-scale phylogenetic architecture of a complex bacterial community. Nature 430, 551–554. 10.1038/nature02649 15282603

[B4] Alcaraz L. D. Belda-Ferre P. Cabrera-Rubio R. Romero H. Simón-Soro A. Pignatelli M.. (2012). Identifying a healthy oral microbiome through metagenomics. Clin. Microbiol. Infect. 18, 54–57. 10.1111/j.1469-0691.2012.03857.x 22647051

[B5] Altschul S. F. Gish W. Miller W. Myers E. W. Lipman D. J. (1990). Basic local alignment search tool. J. Mol. Biol. 215, 403–410. 10.1016/S0022-2836(05)80360-2 2231712

[B6] Asnicar F. Weingart G. Tickle T. L. Huttenhower C. Segata N. (2015). Compact graphical representation of phylogenetic data and metadata with GraPhlAn. PeerJ 3:e1029. 10.7717/peerj.1029 26157614 PMC4476132

[B7] Awano S. Ansai T. Takata Y. Soh I. Akifusa S. Hamasaki T.. (2008). Oral health and mortality risk from pneumonia in the elderly. J. Dent. Res. 87, 334–339. 10.1177/154405910808700418 18362314

[B8] Beck J. D. Offenbacher S. (2005). Systemic effects of periodontitis: epidemiology of periodontal disease and cardiovascular disease. J. Periodontol. 76, 2089–2100. 10.1902/jop.2005.76.11-S.2089 16277581

[B9] Camelo-Castillo A. J. Mira A. Pico A. Nibali L. Henderson B. Donos N.. (2015). Subgingival microbiota in health compared to periodontitis and the influence of smoking. Front. Microbiol. 6:119. 10.3389/fmicb.2015.00119 25814980 PMC4356944

[B10] Caporaso J. G. Kuczynski J. Stombaugh J. Bittinger K. Bushman F. D. Costello E. K.. (2010). QIIME allows analysis of high-throughput community sequencing data. Nat. Methods 7, 335–336. 10.1038/nmeth.f.303 20383131 PMC3156573

[B11] Castellarin M. Warren R. L. Freeman J. D. Dreolini L. Krzywinski M. Strauss J.. (2012). Fusobacterium nucleatum infection is prevalent in human colorectal carcinoma. Genome Res. 22, 299–306. 10.1101/gr.126516.111 22009989 PMC3266037

[B12] Chen H. Jiang W. (2014). Application of high-throughput sequencing in understanding human oral microbiome related with health and disease. Front. Microbiol. 5:508. 10.3389/fmicb.2014.00508 25352835 PMC4195358

[B13] Chen Y. Yang F. Lu H. Wang B. Chen Y. Lei D.. (2011). Characterization of fecal microbial communities in patients with liver cirrhosis. Hepatology 54, 562–572. 10.1002/hep.24423 21574172

[B14] Choudhury S. Isais F. S. Lee C. C. (2013). Nonsurgical management of mitral valve endocarditis due to *Cardiobacterium valvarum* in a patient with a ventricular septal defect. J. Clin. Microbiol. 51, 1996–1997. 10.1128/JCM.00480-13 23576538 PMC3716095

[B15] Ciric L. Pratten J. Wilson M. Spratt D. (2010). Development of a novel multi-triplex qPCR(q)PCR method for the assessment of bacterial community structure in oral populations. Environ. Microbiol. Rep. 2, 770–774. 10.1111/j.1758-2229.2010.00183.x 23766283

[B16] Clarke J. K. (1924). On the bacterial factor in the aetiology of dental caries. Br. J. Exp. Pathol. 5, 141–147.

[B17] De Gannes V. Eudoxie G. Hickey W. J. (2013). Insights into fungal communities in composts revealed by 454-pyrosequencing: implications for human health and safety. Front. Microbiol. 4:164. 10.3389/fmicb.2013.00164 23785368 PMC3682178

[B18] de Soet J. J. Toors F. A. de Graaff J. (1989). Acidogenesis by oral streptococci at different pH values. Caries Res. 23, 14–17. 10.1159/000261148 2920379

[B19] Farrell J. J. Zhang L. Zhou H. Chia D. Elashoff D. Akin D.. (2012). Variations of oral microbiota are associated with pancreatic diseases including pancreatic cancer. Gut 61, 582–588. 10.1136/gutjnl-2011-300784 21994333 PMC3705763

[B20] Featherstone J. D. (2000). The science and practice of caries prevention. J. Am. Dent. Assoc. 131, 887–899. 10.14219/jada.archive.2000.0307 10916327

[B21] Gill S. R. Pop M. Deboy R. T. Eckburg P. B. Turnbaugh P. J. Samuel B. S.. (2006). Metagenomic analysis of the human distal gut microbiome. Science 312, 1355–1359. 10.1126/science.1124234 16741115 PMC3027896

[B22] Gross E. L. Leys E. J. Gasparovich S. R. Firestone N. D. Schwartzbaum J. A. Janies D. A.. (2010). Bacterial 16S sequence analysis of severe caries in young permanent teeth. J. Clin. Microbiol. 48, 4121–4128. 10.1128/JCM.01232-10 20826648 PMC3020839

[B23] Haffajee A. D. Socransky S. S. Patel M. R. Song X. (2008). Microbial complexes in supragingival plaque. Oral Microbiol. Immunol. 23, 196–205. 10.1111/j.1399-302X.2007.00411.x 18402605

[B24] Hagiya H. Kokeguchi S. Ogawa H. Terasaka T. Kimura K. Waseda K.. (2014). Aortic vascular graft infection caused by *Cardiobacterium valvarum*: a case report. J. Infect. Chemother. 20, 804–809. 10.1016/j.jiac.2014.07.008 25242585

[B25] Hajishengallis G. Lamont R. J. (2012). Beyond the red complex and into more complexity: the polymicrobial synergy and dysbiosis (PSD) model of periodontal disease etiology. Mol. Oral Microbiol. 27, 409–419. 10.1111/j.2041-1014.2012.00663.x 23134607 PMC3653317

[B26] Hamady M. Knight R. (2009). Microbial community profiling for human microbiome projects: tools, techniques, and challenges. Genome Res. 19, 1141–1152. 10.1101/gr.085464.108 19383763 PMC3776646

[B27] Han Y. W. (2011). Oral health and adverse pregnancy outcomes - what's next? J. Dent. Res. 90, 289–293. 10.1177/0022034510381905 21041548 PMC3144105

[B28] Hirose H. Hirose K. Isogai E. Miura H. Ueda I. (1993). Close association between *Streptococcus sobrinus* in the saliva of young children and smooth-surface caries increment. Caries Res. 27, 292–297. 10.1159/000261553 8402804

[B29] Hodkinson B. P. Grice E. A. (2015). Next-generation sequencing: a review of technologies and tools for wound microbiome research. Adv. Wound Care (New Rochelle) 4, 50–58. 10.1089/wound.2014.0542 25566414 PMC4281878

[B30] Huson D. H. Mitra S. Ruscheweyh H. J. Weber N. Schuster S. C. (2011). Integrative analysis of environmental sequences using MEGAN4. Genome Res. 21, 1552–1560. 10.1101/gr.120618.111 21690186 PMC3166839

[B31] Ismail Y. Mahendran V. Octavia S. Day A. S. Riordan S. M. Grimm M. C.. (2012). Investigation of the enteric pathogenic potential of oral *Campylobacter concisus* strains isolated from patients with inflammatory bowel disease. PLoS ONE 7:e38217. 10.1371/journal.pone.0038217 22666490 PMC3364211

[B32] Jiang W. Ling Z. Lin X. Chen Y. Zhang J. Yu J.. (2014). Pyrosequencing analysis of oral microbiota shifting in various caries states in childhood. Microb. Ecol. 67, 962–969. 10.1007/s00248-014-0372-y 24504329

[B33] Jiang W. Zhang J. Chen H. (2013). Pyrosequencing analysis of oral microbiota in children with severe early childhood dental caries. Curr. Microbiol. 67, 537–542. 10.1007/s00284-013-0393-7 23743597

[B34] Kanasi E. Dewhirst F. E. Chalmers N. I. Kent R. Jr. Moore A. Hughes C. V.. (2010). Clonal analysis of the microbiota of severe early childhood caries. Caries Res. 44, 485–497. 10.1159/000320158 20861633 PMC2975730

[B35] Keijser B. J. Zaura E. Huse S. M. van der Vossen J. M. Schuren F. H. Montijn R. C.. (2008). Pyrosequencing analysis of the oral microflora of healthy adults. J. Dent. Res. 87, 1016–1020. 10.1177/154405910808701104 18946007

[B36] Kim M. Morrison M. Yu Z. (2011). Evaluation of different partial 16S rRNA gene sequence regions for phylogenetic analysis of microbiomes. J. Microbiol. Methods 84, 81–87. 10.1016/j.mimet.2010.10.020 21047533

[B37] Kumar P. S. Brooker M. R. Dowd S. E. Camerlengo T. (2011). Target region selection is a critical determinant of community fingerprints generated by 16S pyrosequencing. PLoS ONE 6:e20956. 10.1371/journal.pone.0020956 21738596 PMC3126800

[B38] Langille M. G. Zaneveld J. Caporaso J. G. McDonald D. Knights D. Reyes J. A.. (2013). Predictive functional profiling of microbial communities using 16S rRNA marker gene sequences. Nat. Biotechnol. 3, 814–821. 10.1038/nbt.2676 23975157 PMC3819121

[B39] Li J. Helmerhorst E. J. Leone C. W. Troxler R. F. Yaskell T. Haffajee A. D.. (2004). Identification of early microbial colonizers in human dental biofilm. J. Appl. Microbiol. 97, 1311–1318. 10.1111/j.1365-2672.2004.02420.x 15546422

[B40] Li Y. Ge Y. Saxena D. Caufield P. W. (2007). Genetic profiling of the oral microbiota associated with severe early-childhood caries. J. Clin. Microbiol. 45, 81–87. 10.1128/JCM.01622-06 17079495 PMC1828962

[B41] Li Y. Ku C. Y. Xu J. Saxena D. Caufield P. W. (2005). Survey of oral microbial diversity using PCR-based denaturing gradient gel electrophoresis. J. Dent. Res. 84, 559–564. 10.1177/154405910508400614 15914595

[B42] Lindeque P. K. Parry H. E. Harmer R. A. Somerfield P. J. Atkinson A. (2013). Next generation sequencing reveals the hidden diversity of zooplankton assemblages. PLoS ONE 8:e81327. 10.1371/journal.pone.0081327 24244737 PMC3820580

[B43] Ling Z. Kong J. Jia P. Wei C. Wang Y. Pan Z.. (2010). Analysis of oral microbiota in children with dental caries by PCR-DGGE and barcoded pyrosequencing. Microb. Ecol. 60, 677–690. 10.1007/s00248-010-9712-8 20614117

[B44] Loesche W. J. (1979). Clinical and microbiological aspects of chemotherapeutic agents used according to the specific plaque hypothesis. J. Dent. Res. 58, 2404–2412. 10.1177/00220345790580120905 41862

[B45] Loesche W. J. (1986). Role of *Streptococcus mutans* in human dental decay. Microbiol. Rev. 50, 353–380. 3540569 10.1128/mr.50.4.353-380.1986 PMC373078

[B46] Loesche W. J. Rowan J. Straffon L. H. Loos P. J. (1975). Association of *Streptococcus mutants* with human dental decay. Infect. Immun. 11, 1252–1260. 1140847 10.1128/iai.11.6.1252-1260.1975 PMC415207

[B47] Lozupone C. A. Hamady M. Kelley S. T. Knight R. (2007). Quantitative and qualitative beta diversity measures lead to different insights into factors that structure microbial communities. Appl. Environ. Microbiol. 73, 1576–1585. 10.1128/AEM.01996-06 17220268 PMC1828774

[B48] Madison K. M. Bowen W. H. Pearson S. K. Falany J. L. (1991). Enhancing the virulence of *Streptococcus sobrinus* in rats. J. Dent. Res. 70, 38–43. 10.1177/00220345910700010601 1991859

[B49] Marsh P. D. (1994). Microbial ecology of dental plaque and its significance in health and disease. Adv. Dent. Res. 8, 263–271. 7865085 10.1177/08959374940080022001

[B50] Marsh P. D. (2003). Are dental diseases examples of ecological catastrophes? Microbiology 149, 279–294. 10.1099/mic.0.26082-0 12624191

[B51] Marsh P. D. (2006). Dental plaque as a biofilm and a microbial community - implications for health and disease. BMC Oral Health 6:S14. 10.1186/1472-6831-6-S1-S14 16934115 PMC2147593

[B52] Metzker M. L. (2010). Sequencing technologies-the next generation. Nat. Rev. Genet. 11, 31–46. 10.1038/nrg2626 19997069

[B53] Moodley A. Wood N. H. Shangase S. L. (2013). The relationship between periodontitis and diabetes: a brief review. SADJ 68, 260, 262–264. 23971278

[B54] Moreno C. Romero J. Espejo R. T. (2002). Polymorphism in repeated 16S rRNA genes is a common property of type strains and environmental isolates of the genus Vibrio. Microbiology 148, 1233–1239. 10.1099/00221287-148-4-1233 11932467

[B55] Munson M. A. Banerjee A. Watson T. F. Wade W. G. (2004). Molecular analysis of the microflora associated with dental caries. J. Clin. Microbiol. 42, 3023–3029. 10.1128/JCM.42.7.3023-3029.2004 15243054 PMC446285

[B56] Muyzer G. De Waal E. C. Uitterlinden A. G. (1993). Profiling of complex microbial populations by denaturing gradient gel electrophoresis analysis of polymerase chain reaction-amplified genes coding for 16S rRNA. Appl. Environ. Microbiol. 59, 695–700. 7683183 10.1128/aem.59.3.695-700.1993 PMC202176

[B57] Neu J. Lorca G. Kingma S. D. Triplett E. W. (2010). The intestinal microbiome: relationship to type 1 diabetes. Endocrinol. Metab. Clin. North Am. 39, 563–571. 10.1016/j.ecl.2010.05.008 20723820

[B58] Nyvad B. Crielaard W. Mira A. Takahashi N. Beighton D. (2013). Dental caries from a molecular microbiological perspective. Caries Res. 47, 89–102. 10.1159/000345367 23207320

[B59] Obata J. Takeshita T. Shibata Y. Yamanaka W. Unemori M. Akamine A.. (2014). Identification of the microbiota in carious dentin lesions using 16S rRNA gene sequencing. PLoS ONE 9:e103712. 10.1371/journal.pone.0103712 25083880 PMC4118920

[B60] Papaioannou W. Gizani S. Haffajee A. D. Quirynen M. Mamai-Homata E. Papagiannoulis L. (2009). The microbiota on different oral surfaces in healthy children. Oral Microbiol. Immunol. 24, 183–189. 10.1111/j.1399-302X.2008.00493.x 19416446

[B61] Paster B. J. Boches S. K. Galvin J. L. Ericson R. E. Lau C. N. Levanos V. A.. (2001). Bacterial diversity in human subgingival plaque. J. Bacteriol. 183, 3770–3783. 10.1128/JB.183.12.3770-3783.2001 11371542 PMC95255

[B62] Paster B. J. Olsen I. Aas J. A. Dewhirst F. E. (2006). The breadth of bacterial diversity in the human periodontal pocket and other oral sites. Periodontol 2000 42, 80–87. 10.1111/j.1600-0757.2006.00174.x 16930307

[B63] Paul D. Kumbhare S. V. Mhatre S. S. Chowdhury S. P. Shetty S. A. Marathe N. P.. (2016). Exploration of microbial diversity and community structure of lonar lake: the only hypersaline meteorite crater lake within basalt rock. Front. Microbiol. 6:1553. 10.3389/fmicb.2015.01553 26834712 PMC4722114

[B64] Peterson S. N. Snesrud E. Liu J. Ong A. C. Kilian M. Schork N. J.. (2013). The dental plaque microbiome in health and disease. PLoS ONE 8:e58487. 10.1371/journal.pone.0058487 23520516 PMC3592792

[B65] Preza D. Olsen I. Aas J. A. Willumsen T. Grinde B. Paster B. J. (2008). Bacterial profiles of root caries in elderly patients. J. Clin. Microbiol. 46, 2015–2021. 10.1128/JCM.02411-07 18385433 PMC2446847

[B66] Preza D. Olsen I. Willumsen T. Boches S. K. Cotton S. L. Grinde B.. (2009). Microarray analysis of the microflora of root caries in elderly. Eur. J. Clin. Microbiol. Infect. Dis. 28, 509–517. 10.1007/s10096-008-0662-8 19039610 PMC2713778

[B67] Pushalkar S. Mane S. P. Ji X. Li Y. Evans C. Crasta O. R.. (2011). Microbial diversity in saliva of oral squamous cell carcinoma. FEMS Immunol. Med. Microbiol. 61, 269–277. 10.1111/j.1574-695X.2010.00773.x 21205002 PMC3078631

[B68] Ramette A. (2007). Multivariate analyses in microbial ecology. FEMS Microbiol. Ecol. 62, 142–160. 10.1111/j.1574-6941.2007.00375.x 17892477 PMC2121141

[B69] Reading N. C. Sperandio V. (2006). Quorum sensing: the many languages of bacteria. FEMS Microbiol. Lett. 254, 1–11. 10.1111/j.1574-6968.2005.00001.x 16451172

[B70] Schloss P. D. Gevers D. Westcott S. L. (2011). Reducing the effects of PCR amplification and sequencing artifacts on 16S rRNA-based studies. PLoS ONE 6:e27310. 10.1371/journal.pone.0027310 22194782 PMC3237409

[B71] Schultz-Haudt S. Bruce M. A. Bibby B. G. (1954). Bacterial factors in nonspecific gingivitis. J. Dent. Res. 33, 454–458. 10.1177/00220345540330040301 13184032

[B72] Schulze-Schweifing K. Banerjee A. Wade W. G. (2014). Comparison of bacterial culture and 16S rRNA community profiling by clonal analysis and pyrosequencing for the characterization of the dentine caries-associated microbiome. Front. Cell. Infect. Microbiol. 4:164. 10.3389/fcimb.2014.00164 25429361 PMC4228914

[B73] Segata N. Izard J. Waldron L. Gevers D. Miropolsky L. Garrett W. S.. (2011). Metagenomic biomarker discovery and explanation. Genome Biol. 12:R60. 10.1186/gb-2011-12-6-r60 21702898 PMC3218848

[B74] Selwitz R. H. Ismail A. I. Pitts N. B. (2007). Dental caries. Lancet 369, 51–59. 10.1016/S0140-6736(07)60031-2 17208642

[B75] Shade A. Handelsman J. (2012). Beyond the Venn diagram: the hunt for a core microbiome. Environ. Microbiol. 14, 4–12. 10.1111/j.1462-2920.2011.02585.x 22004523

[B76] Shannon P. Markiel A. Ozier O. Baliga N. S. Wang J. T. Ramage D.. (2003). Cytoscape: a software environment for integrated models of biomolecular interaction networks. Genome Res. 13, 2498–2504. 10.1101/gr.1239303 14597658 PMC403769

[B77] Signori C. N. Thomas F. Enrich-Prast A. Pollery R. C. Sievert S. M. (2014). Microbial diversity and community structure across environmental gradients in Bransfield Strait, Western Antarctic Peninsula. Front. Microbiol. 5:647. 10.3389/fmicb.2014.00647 25566198 PMC4267279

[B78] Siqueira J. F. Jr. Rôças I. N. (2009). Diversity of endodontic microbiota revisited. J. Dent. Res. 88, 969–981. 10.1177/0022034509346549 19828883

[B79] Tanner A. C. Mathney J. M. Kent R. L. Chalmers N. I. Hughes C. V. Loo C. Y.. (2011). Cultivable anaerobic microbiota of severe early childhood caries. J. Clin. Microbiol. 49, 1464–1474. 10.1128/JCM.02427-10 21289150 PMC3122858

[B80] Tilg H. (2010). Obesity, metabolic syndrome, and microbiota: multiple interactions. J. Clin. Gastroenterol. 44, S16–S18. 10.1097/MCG.0b013e3181dd8b64 20535027

[B81] Tsai C. Y. Tang C. Y. Tan T. S. Chen K. H. Liao K. H. Liou M. L. (2016). Subgingival microbiota in individuals with severe chronic periodontitis. J. Microbiol. Immunol. Infect. [Epub ahead of print]. 10.1016/j.jmii.2016.04.007 27262209

[B82] Tschöp M. H. Hugenholtz P. Karp C. L. (2009). Getting to the core of the gut microbiome. Nat. Biotechnol. 27, 344–346. 10.1038/nbt0409-344 19352371

[B83] Turnbaugh P. J. Gordon J. I. (2009). The core gut microbiome, energy balance and obesity. J. Physiol. 587, 4153–4158. 10.1113/jphysiol.2009.174136 19491241 PMC2754355

[B84] Turnbaugh P. J. Ley R. E. Hamady M. Fraser-Liggett C. M. Knight R. Gordon J. I. (2007). The human microbiome project: exploring the microbial part of ourselves in a changing world. Nature 449, 804–810. 10.1038/nature06244 17943116 PMC3709439

[B85] Vanerková M. Zaloudíková B. Nemcová E. Terasaka T. Kimura K. Waseda K.. (2010). Detection of *Cardiobacterium valvarum* in a patient with aortic valve infective endocarditis by broad-range PCR. J. Med. Microbiol. 59, 231–234. 10.1099/jmm.0.012948-0 19797468

[B86] Wade W. G. (2013). The oral microbiome in health and disease. Pharmacol. Res. 69, 137–143. 10.1016/j.phrs.2012.11.006 23201354

[B87] Wall-Manning G. M. Sissons C. H. Anderson S. A. Lee M. (2002). Checkerboard DNA-DNA hybridisation technology focused on the analysis of Gram-positive cariogenic bacteria. J. Microbiol. Methods 51, 301–311. 10.1016/S0167-7012(02)00106-9 12223290

[B88] Xu H. Hao W. Zhou Q. Wang W. Xia Z. Liu C.. (2014). Plaque bacterial microbiome diversity in children younger than 30 months with or without caries prior to eruption of second primary molars. PLoS ONE 9:e89269. 10.1371/journal.pone.0089269 24586647 PMC3938432

[B89] Yang F. Zeng X. Ning K. Liu K. L. Lo C. C. Wang W.. (2012). Saliva microbiomes distinguish caries-active from healthy human populations. ISME J. 6, 1–10. 10.1038/ismej.2011.71 21716312 PMC3246229

[B90] Yarza P. Richter M. Peplies J. Euzeby J. Amann R. Schleifer K. H.. (2008). The all-species living tree project: a 16S rRNA-based phylogenetic tree of all sequenced type strains. Syst. Appl. Microbiol. 31, 241–250. 10.1016/j.syapm.2008.07.001 18692976

[B91] Yoo J. Y. Rho M. You Y. A. Kwon E. J. Kim M. H. Kym S.. (2016). 16S rRNA gene-based metagenomic analysis reveals differences in bacteria-derived extracellular vesicles in the urine of pregnant and non-pregnant women. Exp. Mol. Med. 48:e208. 10.1038/emm.2015.110 26846451 PMC4892867

[B92] Zaura E. (2012). Next-generation sequencing approaches to understanding the oral microbiome. Adv. Dent. Res. 24, 81–85. 10.1177/0022034512449466 22899686

[B93] Zaura E. Keijser B. J. Huse S. M. Crielaard W. (2009). Defining the healthy “core microbiome” of oral microbial communities. BMC Microbiol. 9:259. 10.1186/1471-2180-9-259 20003481 PMC2805672

