# Supplementary figures and images for: Bacterial Diversity and Community Structure of Supragingival Plaques in Adults with Dental Health or Caries Revealed by 16S Pyrosequencing
Source: Front Microbiol. 2016 Jul 22;7:1145. doi: 10.3389/fmicb.2016.01145 (PMC4956651; doi:10.3389/fmicb.2016.01145)

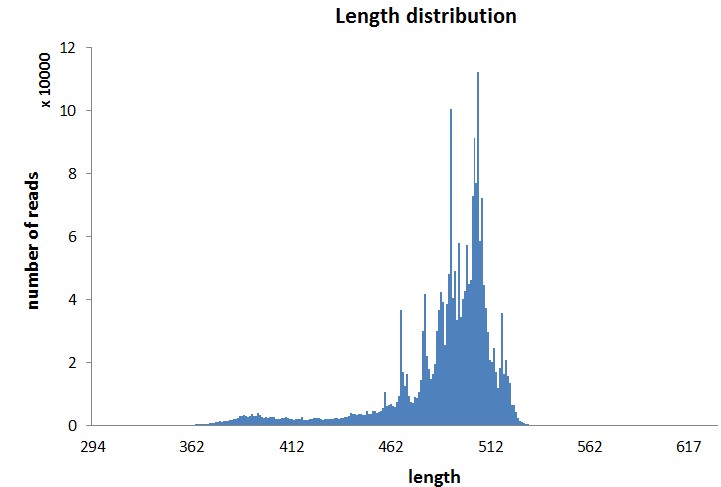

Supplement: Figure S1 — Length distribution of sequences determined by 454 pyrosequencing. [file Image1.JPEG]

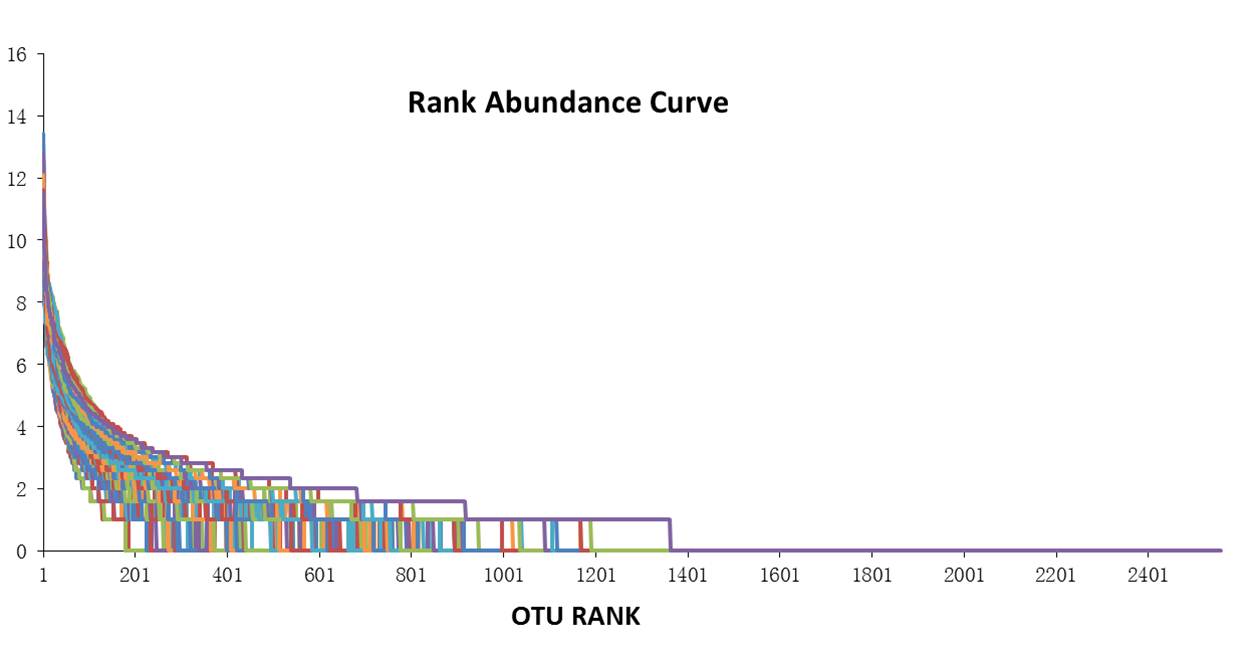

Supplement: Figure S2 — Rank abundance curves for all OTUs. [file Image2.JPEG]

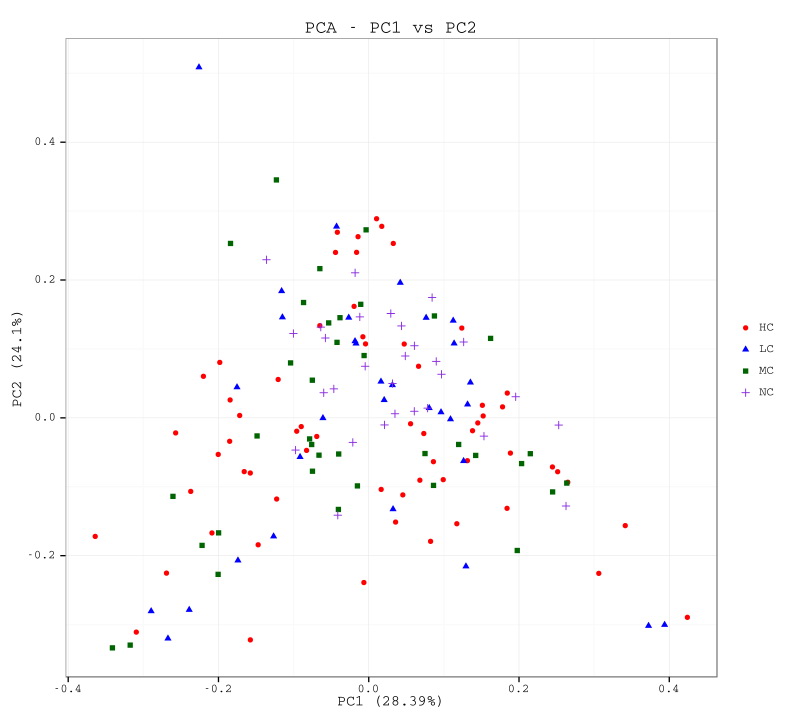

Supplement: Figure S3 — Principal component analysis (PCA) at the genus level. [file Image3.JPEG]

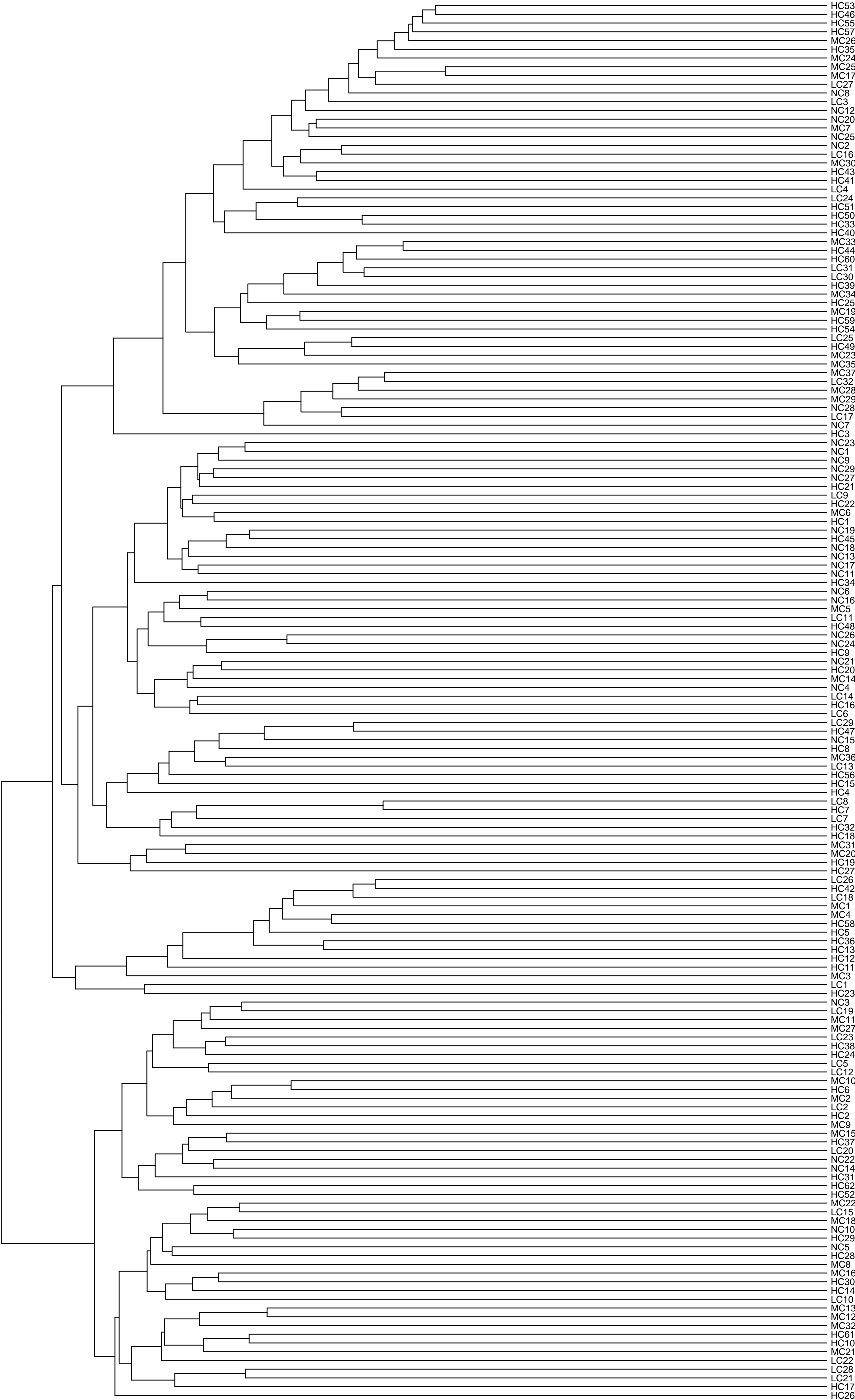

Supplement: Figure S4 — UPGMA hierarchical clustering analysis. [file Image4.PDF]

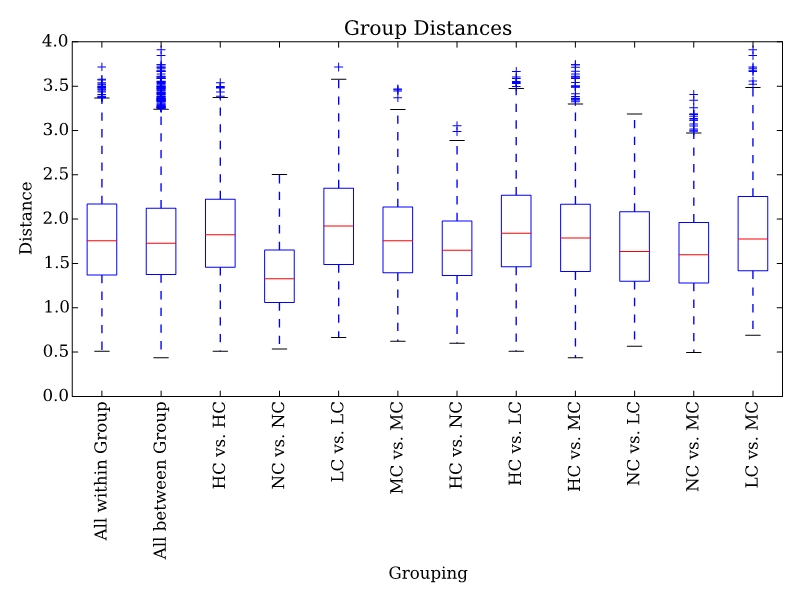

Supplement: Figure S5 — Box-and-whiskers plot of the Unifrac distance. [file Image5.JPEG]

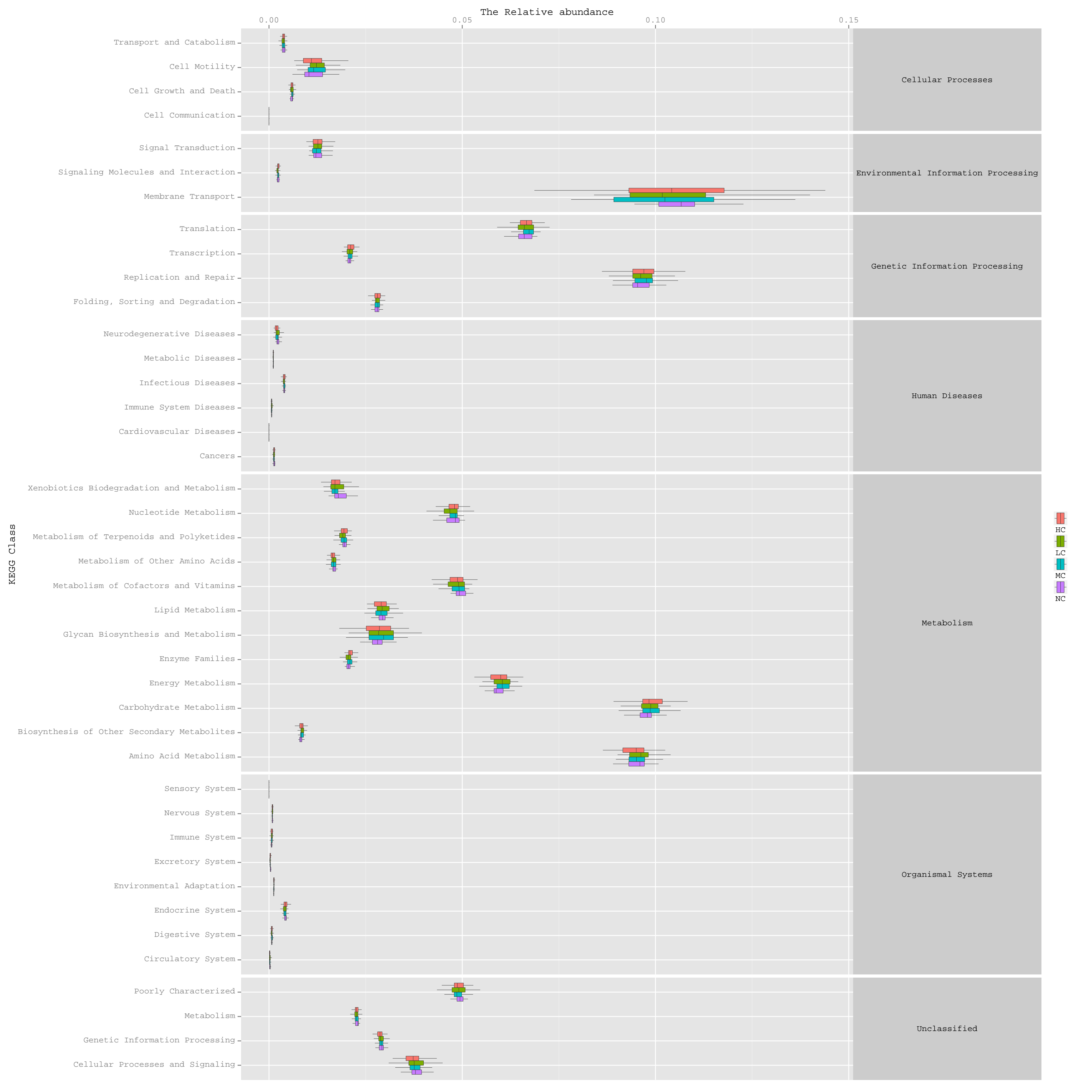

Supplement: Figure S6 — Bacterial function prediction by PICRUSt analysis. [file Image6.PDF]
